# Periodontal bacterial colonization in synovial tissues exacerbates collagen-induced arthritis in B10.RIII mice

**DOI:** 10.1186/s13075-016-1056-4

**Published:** 2016-07-12

**Authors:** Sasanka Chukkapalli, Mercedes Rivera-Kweh, Prashasnika Gehlot, Irina Velsko, Indraneel Bhattacharyya, S. John Calise, Minoru Satoh, Edward K. L. Chan, Joseph Holoshitz, Lakshmyya Kesavalu

**Affiliations:** Department of Periodontology and Oral Biology, College of Dentistry, University of Florida Gainesville, Gainesville, FL 32610 USA; Department of Internal Medicine, University of Michigan School of Medicine, Ann Arbor, Michigan USA; Department of Oral & Maxillofacial Diagnostic Sciences, College of Dentistry, University of Florida, Gainesville, Florida USA; Departments of Oral Biology, College of Dentistry, University of Florida, Gainesville, Florida USA; Division of Rheumatology and Clinical Immunology, College of Medicine, University of Florida, Gainesville, Florida USA; Department of Clinical Nursing, University of Occupational and Environmental Health, Kitakyushu, Fukuoka Japan

**Keywords:** Rheumatoid arthritis, Periodontal disease, Periodontal bacteria, *Porphyromonas gingivalis*, B10.RIII mice, Collagen-induced arthritis

## Abstract

**Background:**

It has been previously hypothesized that oral microbes may be an etiological link between rheumatoid arthritis (RA) and periodontal disease. However, the mechanistic basis of this association is incompletely understood. Here, we investigated the role of periodontal bacteria in induction of joint inflammation in collagen-induced arthritis (CIA) in B10.RIII mice.

**Methods:**

CIA-prone B10.RIII mice were infected orally with a polybacterial mixture of *Porphyromonas gingivalis*, *Treponema denticola*, and *Tannerella forsythia* for 24 weeks before induction of CIA. The ability of polybacterial mixture to colonize the periodontium and induce systemic response, horizontal alveolar bone resorption in infected B10.RIII mice was investigated. Arthritis incidence, severity of joint inflammation, pannus formation, skeletal damage, hematogenous dissemination of the infection, matrix metalloproteinase 3 (MMP3) levels, and interleukin-17 expression levels were evaluated.

**Results:**

B10.RIII mice had gingival colonization with all three bacteria, higher levels of anti-bacterial immunoglobulin G (IgG) and immunoglobulin M (IgM) antibodies, significant alveolar bone resorption, and hematogenous dissemination of *P. gingivalis* to synovial joints. Infected B10.RIII mice had more severe arthritis, and higher serum matrix metalloproteinase 3 levels and activity. Histopathological analysis showed increased inflammatory cell infiltration, destruction of articular cartilage, erosions, and pannus formation. Additionally, involved joints showed had expression levels of interleukin-17.

**Conclusion:**

These findings demonstrate that physical presence of periodontal bacteria in synovial joints of B10.RIII mice with collagen-induced arthritis is associated with arthritis exacerbation, and support the hypothesis that oral bacteria, specifically *P. gingivalis*, play a significant role in augmenting autoimmune arthritis due to their intravascular dissemination to the joints.

**Electronic supplementary material:**

The online version of this article (doi:10.1186/s13075-016-1056-4) contains supplementary material, which is available to authorized users.

## Background

Rheumatoid arthritis (RA) and periodontal disease (PD) are epidemiologically associated with each other [1–3], but the mechanistic basis of this association is unclear. It is known, however, that the pathogeneses of both conditions involve TNFα, T helper (Th)17 cells, and osteoclast-mediated bone damage [4, 5]. Similar to RA, the susceptibility to PD and its severity depend on overlapping environmental and genetic factors, as both RA and PD have been shown to be associated with the human leukocyte antigen (HLA) shared epitope [6–8] and cigarette smoking has been shown to increase disease risk in both conditions [9].

It has long been hypothesized that oral microbes may be an etiological link between PD and RA in humans [10, 11]. For example, in Native American patients with RA and their relatives, antibodies to the periodontal bacteria *Porphyromonas gingivalis* (*P. gingivalis*) were found to be associated with anti-citrullinated protein antibodies, suggesting that *P. gingivalis* may be breaking the immune tolerance towards citrullinated antigens [12]. However, there is no direct causative evidence to support this or other proposed mechanisms for the long-observed association between human RA and PD.

It is worth noting that heat-killed *P. gingivalis* have been previously proposed as a “priming” inflammatory agent linking experimental PD and arthritis in a rat model [13]. That study demonstrated that the presence of extra-synovial chronic inflammatory lesions, induced by heat-killed *P. gingivalis*, promoted the induction and severity of experimental arthritis. Further, the same group [14] tried to assess the influence of preexisting PD on induction and severity of collagen antibody-induced arthritis (CIA) in mice. Using pristine-induced arthritis (PIA) Trombone et al. [15] described the clinical association between RA and PD in the acute inflammatory reactivity maximum (AIRmax) mice. More recently, Marchesan and associates have demonstrated strain-specific immune system divergence following infection with different strains of *P. gingivalis* [16]. Moreover, *P. gingivalis* orally-infected DBA/1 mice with CIA had more severe arthritis associated with activation of Th17-related pathways [16], suggesting that this T cell subset may directly contribute to the observed association between arthritis and PD. However, given the generalized nature of the Th17 activation, the mechanistic basis of the predilection of *P. gingivalis*-infected mice to trigger a tissue-specific inflammatory process in the joints is still unclear.

To better elucidate the mechanistic basis of the association between RA and PD, here we have taken a different experimental approach by focusing on CIA in B10.RIII mice, which are genetically susceptible to collagen type II (CII)-induced arthritis. As periodontal disease always results from the dysbiotic interaction between the oral microbiota and host immunity we have chosen the most representative microorganisms that have been established as periodontal pathogens. We induced periodontal disease in these B10.RIII mice by chronic gingival infection with a combination of *P. gingivalis*, *T. denticola* and *T. forsythia* for 24 weeks [17, 18]. Our data demonstrate that mice chronically infected with these PD-causing bacteria experienced aggravated clinical signs of CIA with increased metalloproteinase activity, intense immune-based inflammatory cellular infiltration, and enhanced destruction of articular cartilage and bone. Importantly, a fluorescence *in situ* hybridization technique revealed dissemination of the periodontal bacteria to the synovial tissues.

These findings substantiate a previously unappreciated mechanism of cause-effect relationship between periodontal infection and arthritis, and support the hypothesis that PD-causing bacteria, specifically *P. gingivalis*, may contribute to the susceptibility and severity of inflammatory arthritis due to their tropism to synovial tissues, where they may contribute to intensifying the inflammatory process.

## Methods

### Microbial strains and inocula

*P. gingivalis* FDC 381, *T. denticola* ATCC 35404, and *T. forsythia* ATCC 43037 were used in this study and were routinely cultured anaerobically at 37 °C as described previously [17–19]. Bacterial inocula were prepared and used for gingival infection of mice by oral lavage as described previously [17, 18, 20, 21].

### Mouse infection and oral plaque sampling

**Fig. 1:**
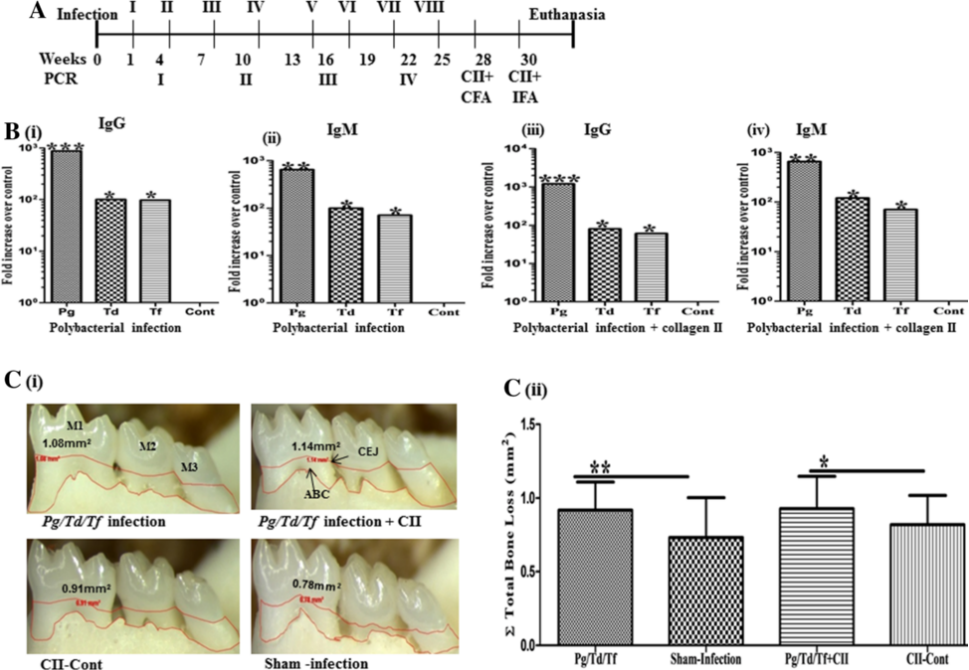
Establishment of periodontal disease in B10.RIII mice. **a** Schematic diagram of the experimental design. **b (i)**, **(ii)** Polybacterial-infected mice IgG, IgM systemic immune response against *P. gingivalis* (*Pg*), *T. denticola* (*Td*)*,* and *T. forsythia* (*Tf*), respectively; **(iii)**, **(iv)** polybacterial-infected + collagen type II (CII)-immunized mice IgG, IgM systemic immune response against *Pg, Td*, and *Tf*, respectively. **c (i)** Representative images of horizontal alveolar bone resorption area in mandibular surfaces of B10.RIII mice; **(ii)** total alveolar bone resorption in B10.RIII mice. Each *bar* indicates the mean alveolar bone resorption for three molars in each quadrant. Three bacteria were used in oral infection for 24 weeks. *N* = 10 in each group. *P < 0.05, ***P < 0.001. *Cont* control

The polybacterial oral infection and sampling methodology were done as described previously [17, 18]. Briefly, six-week-old male B10.RIII mice (The Jackson Laboratories, Bar Harbor, ME, USA) were kept in groups and housed in microisolator plastic cages. Mice were randomly distributed into four groups; polybacterial infection alone (group I; *n* = 10), polybacterial infection and immunization with complete Freund's adjuvant (CFA)/CII and incomplete Freund’s adjuvant (IFA)/CII (group II; *n* = 10), immunization with CFA/CII and IFA/CII alone (group III; *n* = 10), and sham-infection control (group IV; *n* = 10) (Table 1). B10.RIII mice were administered kanamycin (500 μg/ml) daily for 3 days in the drinking water and the mouse oral cavity was rinsed with 0.12 % chlorhexidine gluconate (Peridex: 3M ESPE Dental Products, St. Paul, MN, USA) mouth rinse to reduce endogenous murine microorganisms and to enhance subsequent colonization of human periodontal bacteria [17]. The concentration of each bacterium used for infection was determined quantitatively, and the organisms were resuspended in reduced transport fluid at 1 × 10^10^ bacteria per ml. Bacteria were then mixed with an equal volume of sterile 4 % (wt/vol) low-viscosity carboxymethylcellulose (CMC; Sigma-Aldrich, St. Louis, MO, USA) and polybacterial inocula used for gingival infection were administered (10^9^ cells in 0.2 ml) for 4 consecutive days per week on 8 alternate weeks to mimic chronic exposure during 24 weeks of the infection period (Fig. 1a). Mice swallowed the bacterial inoculum, which ends up in the gut where there is a chance of gut infection-induced inflammation and possible systemic entry or a potential route for the induction of experimental arthritis. Sham-infected control mice received sterile 4 % carboxymethylcellulose (CMC) only. Gingival plaque samples were collected at 3 days post-infection by swabbing the gingival surface of the mice, especially the teeth and surrounding gingival tissue, using a sterile veterinary cotton swab with a head width of 2.6 mm.

**Table 1:** Distribution of B10.RIII mice groups, polybacterial infection and collagen administration in the induction of collagen-induced arthritis

Group	Bacterial infection (24 weeks)	Collagen-induced arthritis (25–28 weeks)	Number of mice
I	*Pg/Td/Tf*	----------------------	10
II	*Pg/Td/Tf*	CII + CFA primary	10
		CII + IFA booster	
III	Sham infection	CII + CFA primary	10
		CII + IFA booster	
IV	Sham infection	----------------------	10

*Pg* indicates *P. gingivalis*; *Td* indicates *T. denticola*; *Tf* indicates *T. forsythia. CII* collagen type II, CFA complete Freund’s adjuvant, *IFA* incomplete Freund’s adjuvant

### Induction and clinical evaluation of collagen-induced arthritis

For induction of CIA, pre-dissolved liquid bovine type II collagen (bCII; 2 mg/ml, Chondrex LLC, Redmond, WA, USA) was emulsified with an equal volume of CFA or IFA (Chondrex LLC). After 24 weeks of infection, mice were immunized intradermally at the base of the tail with 0.1 ml of emulsion containing 100 μg of CII and CFA. Three weeks after priming (day 21), the mice were boosted with 0.1 ml of bovine CII (100 μg) emulsified in an equal volume of IFA. All mice were monitored three times a week by the same person blinded to the groups and arthritis severity was assessed using criteria as follows: 0 = no swelling or redness (normal); 1 = mild erythema or swelling of the wrist or ankle or erythema and swelling of one digit; 2 = moderate erythema and swelling of the wrist or ankle or more than three inflamed digits; 3 = severe erythema and swelling of the wrist or ankle; and 4 = complete erythema and swelling of the wrist and ankle including all digits [22–24]. A paw was considered arthritic when the individual paw score was >1 and severe arthritis was defined as an arthritis score >3 for the purpose of comparing data between groups.

### Detection of *P. gingivalis*, *T. denticola,* and *T. forsythia* genomic DNA in oral plaque

Colony PCR was performed with gingival plaque samples obtained after every infection in a Bio-Rad thermal cycler using 16S rRNA gene species-specific oligonucleotide primers. *P. gingivalis*: 5′-TGTAGATGACTGATGGTGAAAACC-3′ (forward), 5′-ACGTCATCCCCACCTTCCTC-3′ (reverse); *T. denticola* 5′-TAATACCGAATGTGCTCATTTACAT-3′ (forward), 5′-CTGCCATATCTCTATGTCATTGCTCTT-3′ (reverse); and *T. forsythia* 5′-AAAACAGGGGTTCCGCATGG-3′ (forward), 5′-TTCACCGCGGACTTAACAGC-3′ (reverse). Genomic DNA extracted from these three strains served as positive controls and PCR performed with no template DNA served as negative control. PCR was performed following the conditions described previously [17], PCR products were separated by 1.5 % agarose gel electrophoresis and the bands were visualized using the UVP BioDoc-It Imaging System (UVP, Upland, CA, USA).

### Pathogen-specific immune response in mice

Sera from infected and control B10.RIII mice were used to determine immunoglobulin G (IgG) and IgM antibody concentrations against whole cells (formalin-killed) of *P. gingivalis*, *T. denticola*, and *T. forsythia* by ELISA [18, 20].

### Morphometric analysis of alveolar bone resorption

The horizontal alveolar bone resorption (ABR) area and the presence of periodontal intra-bony defects were measured by histomorphometry as described previously [17, 21].

### Detection of bacterial genomic DNA in internal organs

Heart, aorta, liver, pancreas, spleen, kidney, lung and joints/synovial tissues were harvested after euthanasia of infected/sham-infected mice. Tissues were transferred to the laboratory in vials containing reduced transport fluid and were stored at –80 °C until further use. Later they were thawed, homogenized using a mechanical tissue disruptor (TissueRuptor®, QIAGEN, Valencia, CA, USA) and genomic DNA extracted using QIAGEN DNeasy blood and tissue kit (QIAGEN) per the protocol described in the kit. Subsequently PCR was performed using 16S rRNA primers specific for each of the infection bacteria as described previously [19].

### Detection of bacteria by fluorescence *in situ* hybridization (FISH)

FISH was performed on formalin-fixed paraffin-embedded ankle tissue sections using oligonucleotide probes labeled with Alexa Fluor 568 (Invitrogen, Carlsbad, CA, USA) that are specific for 16S rRNA of *P. gingivalis*, (5′-CAATACTCGTATCGCCCGTTATTC-3′), ’*T. denticola* 5′- CATGACTACCGTCATCAAAGAAGC-3′), or *T. forsythia* (5′-CGTATCTCATTTTATTCCCCTGTA-3′) [24–26] 16S rRNA. The protocol was performed as previously described [24].

### In vivo molecular imaging/tomography of mice

An intravenous injection of fluorescent imaging agent (MMP Sense 750 FAST, PerkinElmer, Waltham, MA, USA) specific for MMP3 was given at a recommended dose of 2 nmol/100 μl per mouse (as per the manufacturer’s instruction) to measure the progression of arthritis [27]. MMP Sense FAST is an activatable fluorescent imaging compound that is optically silent upon injection but produces fluorescent signal after cleavage by disease-related MMPs. The signals emitted were detected using the IVIS system (Caliper Life Sciences, MA, USA) which is an optimized set of high-efficiency filters and spectral un-mixing algorithms to measure the light emission across the blue to near infrared wavelength region.

### Histological examination

Hind limbs together with the overlying skin from mice were excised at the termination of the experiment, and fixed in 10 % neutral buffered formalin. Later they were decalcified in 10 % ethylenediaminetetraacetic acid (EDTA) and embedded in paraffin. Serial sections (5 μm) were made and stained with hematoxylin/eosin and evaluated for synovial inflammation, pannus formation, and bone erosion [27]. Histopathological analysis was conducted by a pathologist who was blinded to the experimental study groups. Pannus formation was quantified based on a scoring criterion as follows: a score of 0 was given to no pannus formation; 1 for minimal formation; and 2 for definitive formation. Skeletal damage was quantified as 0 for none; 1 for one or two foci seen; 2 for multiple areas seen; and 3 for extensive damage among all surface areas. Synovial inflammation was scored based on scoring criteria [28] as follows, 0: no hyperplasia or inflammation; 1: slight hyperplasia with scattered acute inflammation; 2: multiple foci of inflammation predominantly with neutrophils; and 3: strong inflammation with inflammatory cell infiltration.

### Matrix metalloproteinase 3 levels in serum from infected mice

Serum from B10.RIII mice (four groups; *n* = 6) was used to detect levels of MMP3 using the commercial serum MMP3 ELISA kit (Sigma-Aldrich Co, USA).

### Immunohistochemical analysis

Tissues were fixed in 10 % buffered formalin, decalcified, embedded in paraffin wax and cut to 5 μm thickness, mounted on slides and air-dried at room temperature. Sections were deparaffinized, rehydrated and incubated with 3 % hydrogen peroxide in methanol for 15 minutes at room temperature to eliminate endogenous peroxidase activity. Antigen retrieval was carried out at 95 °C for 30 minutes by placing the slides in 0.01 M sodium citrate buffer (pH 6.0). The slides were then incubated with a primary rabbit polyclonal antibody for IL-17 (Abcam #ab79056) at 4 °C overnight. For immune detection, the avidin-biotin complex method was performed according to the manufacturer’s instructions. Color development was achieved with 3, 3′-diaminobenzidine, which renders positive cells brown. Photographs were taken using an Olymbus BX-60 upright microscope (Center for live-cell imaging, UM-Michigan, USA).

### Immunofluorescence

For immunofluorescence quantification, slides were stained with a primary rabbit polyclonal antibody for IL-17 as above, followed by secondary donkey anti-rabbit IgG antibody conjugated to Alexa Fluor® 594 (Abcam #ab150076) and stored at 4 °C until use. 4′ 6-diamidino-2-phenylindole (DAPI) was used for nuclei staining. Quantification was done by a Biotek Cytation 5 instrument, using the Gen5Image + software (Biotek), which allows for calculation of the percentage of nucleated (DAPI-positive) cells with cytoplasmic IL-17.

### Statistical analysis

Group measures are expressed as mean plus SEM. Statistical analyses were performed using the two-tailed Student’s *t* test with GraphPad Prism 5 (GraphPad, San Diego, CA, USA), and *P* < 0.05 was considered statistically significant (*P < 0.05; **P < 0.01; ***P < 0.001). For multiple-group comparisons, one-way analysis of variance (*P* < 0.05) with Bonferroni’s multiple-comparison was performed posttest.

## Results

### Gingival colonization and antibody response to periodontal-disease-associated polybacteria in mice

To investigate the cause-effect relationship between PD and inflammatory arthritis, we sought to determine the effects of known human PD-associated polybacteria on the severity of CIA. Because DBA/1, the most commonly used mouse strain for the arthritis model, were resistant to oral colonization despite prolonged attempts to infect them over a 24-week period (data not shown), we chose to focus on B10.RIII mice. The B10.RIII mice were susceptible to such colonization. As shown in Table 2, B10.RIII infected mice had colonization (second and sixth infection cycle) with three periodontal pathogens, *P. gingivalis*, *T. denticola,* and *T. forsythia*. All B10.RIII mice in the polybacteria-infected group (Fig. 1b (i) and (iii)) developed significantly elevated IgG antibody to *P. gingivalis* (*P* < 0.001)*, T. denticola* (*P* < 0.05), and *T. forsythia* (*P* < 0.05) compared to the levels in sham-infected mice. However, anti-*P. gingivalis* IgG antibody titers were higher than anti-*T. denticola* and anti-*T. forsythia* IgG antibody levels (Fig. 1b (i) and (iii)). Similarly, all B10.RIII mice in the polybacteria-infected group (Fig. 1b (ii) and (iv)) developed significantly elevated IgM antibodies to *P. gingivalis* (*P* < 0.01)*, T. denticola* (*P* < 0.05), and *T. forsythia* (*P* < 0.05) compared to the levels in sham-infected mice and uninfected CIA mice. Thus, B10.RIII mice are susceptible to infection with PD-associated polymicrobial pathogens, and develop antibody response to these pathogens.

**Table 2:** Gingival plaque samples positive for bacterial gDNA identified by PCR

Group	Polybacterial infection	Positive gingival plaque samples (*n* = 10)				
		1 week^a^	2 weeks	4 weeks	6 weeks	8 weeks
I	*Pg/Td/Tf*	NC	7/0/6	NC	7/7/7	NC
II	*Pg/Td/Tf* + collagen	NC	8/0/8	NC	9/7/7	NC
III	Collagen-control	NC	NC	0/0/0	NC	0/0/0
IV	Sham-infected	NC	NC	0/0/0	NC	0/0/0

Total numbers of gingival plaque samples that were collected after infections (1, 2, 4, 6 and 8 weeks) following polymicrobial (*P. gingivalis/T. denticola/T. forsythia* (*Pg/Td/Tf*)) infection and were positive as determined by PCR analysis. ^a^Time points at which gingival plaque samples were collected. The first value corresponds to the number of mice that tested positive for *Pg* genomic DNA, the second value to the number of mice that tested positive for *Td* genomic DNA, and the third value to the number of mice that tested positive for *Tf* genomic DNA at each time point. *NC* not collected (to allow bacterial biofilm to adhere to the gingival surface, invade epithelial cells, and multiply), *Pg/Td/Tf* polybacterial-infected mice, *Pg/Td/Tf + collagen* polybacterial-infected mice administered collagen II, *Collagen-control* mice administered collagen II

### Periodontal-disease-associated bacteria increase periodontal disease and severity of collagen-induced arthritis

The impact of these three bacteria on PD disease severity was investigated next. To this end we determined alveolar bone resorption, the hallmark characteristic of PD, using a morphometric approach. Among the B10.RIII mice the polybacterial-infected mice had a significantly larger (*P* < 0.01) mandibular horizontal ABR area when compared to sham-infected mice (Fig. 1c (i) and (ii)).

**Fig. 2:**
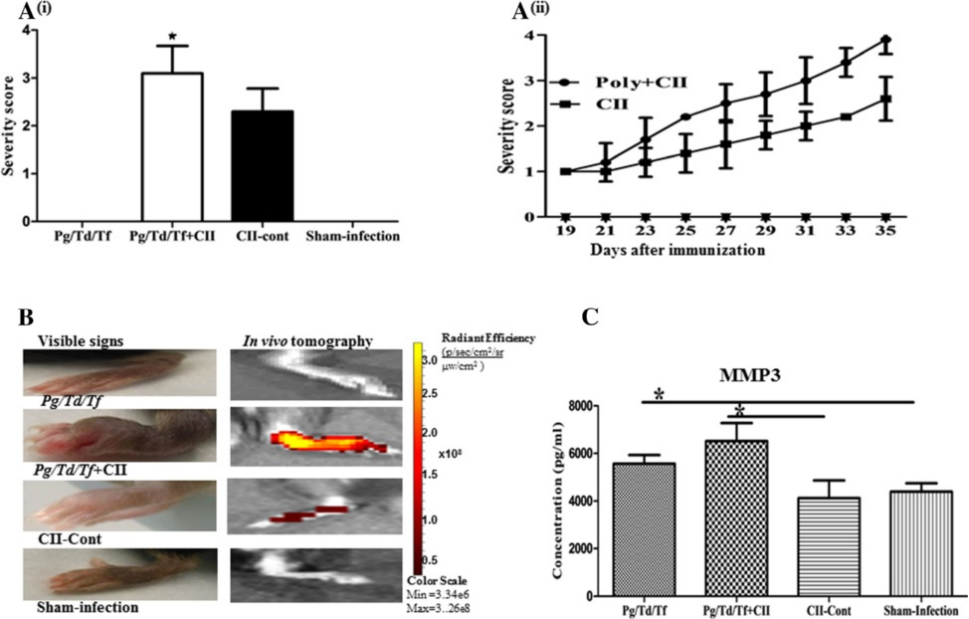
Periodontal infection exacerbates CIA in B10.RIII mice. **a (i)** Visual clinical severity score in B10.RIII mice. Poly + CII group mice were infected with polybacterial infection first followed by primary immunization with collagen type II (*CII*) + complete Freund’s adjuvant followed by booster after 3 weeks with CII + incomplete Freund’s adjuvant. (*n* = 10); **(ii)** increased clinical arthritis scores in the hind paws upon concomitant periodontitis and collagen immunization. **b** Exacerbation of arthritic signs detected by in vivo tomography in B10.RIII mice. Photographs show visible inflammation in hind paws of mice (*left*). IVIS spectrum images show emission of fluorescence signals in mice (*right*). **c** Elevation of serum matrix metalloproteinase 3 (*MMP3*) in a mouse infected with *P. gingivalis/T. denticola/T. forsythia* (*Pg/Td/Tf*). *Bars* show mean ± SD. *n* = 6; *P < 0.05. *Cont* control

We then evaluated the effect of chronic gingival infection on CIA, which was induced in B10.RIII mice after chronic polybacterial infection. As shown in Fig. 2a (i), all (polybacterial-infected and CII-immunized) B10.RIII mice developed greater clinical signs of arthritis in the ankle joints and paws (100 %, 10 out of 10 mice) compared to mice that were polybacterial-infected only (0 %, 0 out of 10 mice). Collagen control B10.RIII mice also developed clinical signs of arthritis in the hind paws (100 %, 10 out of 10 mice). Without CII immunization, none of the polybacterial-infected mice or sham-infected control mice developed clinical signs of arthritis (0 %).

There were robust differences in the polybacteria-infected mice with CIA compared to mice with CIA but without such bacterial infection, in terms of day of arthritis onset, disease incidence, and joint swelling (Fig. 2a (i) and (ii)). In vivo tomography demonstrated the rapid progression of arthritis in polybacterial-infected mice with CIA compared to uninfected mice with CIA (Fig. 2b). There were no signs of arthritis development in polybacterial-infected non-CII immunized mice or in sham-infected mice. While we observed the progression of arthritis with the emission of fluorescence signals specific for arthritis in both the polybacterial-infected plus CII-immunized mice and mice that were CII-immunized only, the degree of intensity and severity was five times greater (measured by emission of radiance) in polybacterial-infected plus CII-immunized mice than in the CII-immunized mice (Fig. 2b). Evaluation of mice for progression of severe arthritis by in vivo tomography correlated with the observed clinical signs of arthritis in polybacterial-infected plus CII-immunized mice (Fig. 2b).

### Effects of polybacterial infection on arthritis biomarkers

We determined the levels of MMP3, an enzyme capable of degrading cartilage and connective tissue in joint tissues in the sera, by ELISA. A significant (*P* < 0.05) elevation in serum MMP3 levels was detected in mice with polybacterial infection with or without CIA compared to sham-infected controls and mice with CIA but without polymicrobial infection (Fig. 2c). In addition, polybacterial-infected mice with CIA had higher MMP3 levels than mice with polybacterial infection without CIA.

### Impact of coincidental periodontal disease and collagen type II immunization on the histopathology of arthritis

**Fig. 3:**
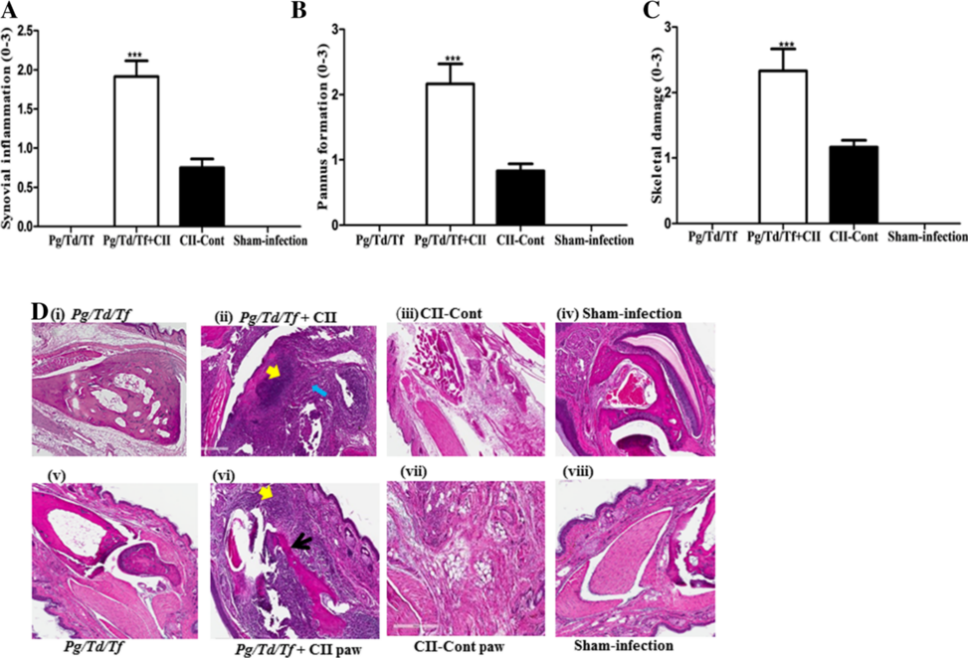
Chronic periodontal infection and concomitant collagen immunization aggravates experimental arthritic pathological change. Histological assessment of ankle joints after immunization with collagen type II (*CII*). **a** Synovial joint inflammation, **b** pannus formation and **c** skeletal damage scores on H&E-stained tissue sections. Data are mean + SEM (scale 0–3) of six mice per group. **d** Histopathological evidence of development of arthritis in B1O.RIII mice. H&E staining of ankle joint tissue from B10.RIII mice (*top row*). H&E staining of paw tissue from B10.RIII mice (*bottom row*). *Yellow arrowheads* indicate inflammatory infiltration; *thick blue arrows* indicate pannus formation and *thick black arrows* indicates cartilage destruction. Original magnification ×20. *N* = 6 in each group. *Cont* control, *Pg/Td/Tf*, *P. gingivalis/T. denticola/T. forsythia*

When we determined that the specific histopathological features of arthritis are affected by periodontal bacterial infection and CII immunization microscopically, we observed a significant increase in synovial inflammation, pannus formation, and skeletal damage when there is coincidental PD and CII immunization in comparison to CII immunization alone, PD alone, or sham-infection (Fig. 3a-c). Further, polymicrobial-infected CIA mice had characteristic inflammatory cell infiltrates and pannus formation in the ankle and paw joints (Fig. 3d (ii); Additional file 1: Figure S1A (i-iii)). These mice also had destruction of cartilage and bone in the joints (Fig. 3d (vi)). In contrast, although uninfected mice with CIA developed minimal inflammatory cell infiltration, pannus and joint destruction could be seen in these mice (Fig. 3d (iii) and (vii); Additional file 1: Figure S1B (i-iii)). There were no observable histopathological signs of arthritis in polybacterial-infected mice that were not immunized with CII (Fig. 3d (i) and (v)), or in sham-infected mice (Fig. 3D (iv) and (viii)).

### Expression of IL-17 in paw tissue

**Fig. 4:**
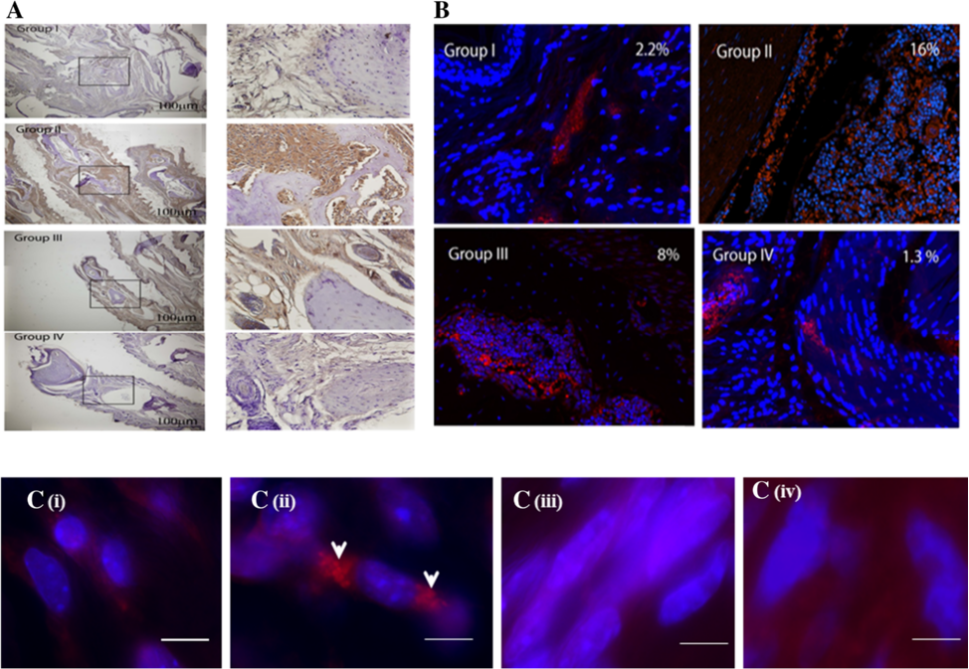
IL-17 expression in synovial tissues. **a** Histological sections of representative B10.RIII mice displaying IL-17 expression in the four mouse groups. *Left column* shows low magnification (×4). *Right column* shows a higher magnification (×40) of the respective *boxed areas* shown in the *left column*. Group II paws had severe joint tissue-invading pannus tissue, which was heavily infiltrated by IL-17-exprescsing cells. **b** Immunofluorescence. Representative images of the joint tissues from different treatment groups. *Blue staining* represents 4′ 6-diamidino-2-phenylindole; *red fluorescence* represents IL-17. The calculated percentage of IL-17-positive cells is shown in the *right upper corner* of each image. Group II paws had much higher abundance of IL-17-expressing cells compared to the three other groups. Group III had an intermediate level of IL-17 abundance**. c (i)**, **(ii)**, **(iii)**, **(iv)** Representative fluorescence *in situ* hybridization images of **c** **(i)** *P. gingivalis/T. denticola/T. forsythia* (*Pg/Td/Tf*)-infected mice with no presence of bacteria, **c (ii)** the presence of *P. gingivalis* (*bright red fluorescence* denoted by *white arrowheads*) in ankle joint tissue of mice infected with *Pg/Td/Tf* along with collagen immunized in B10.RIII mice, **c (iii)** collagen control infected mice with no presence of bacteria, **c (iv)** sham-infected mice with no presence of bacteria*. Scale bar* represents 10 μm

There are increasing reports suggesting that Th17 plays a dominant role in the progression of periodontal disease [29–31] and IL-17A, produced by Th17 and other cells, has previously been described as an inflammatory cytokine, which induces additional cytokines, chemokines, and metalloproteinases that contribute to joint destruction in arthritis [32]. We therefore sought to determine whether expression of IL-17 in tissue could distinguish between the different mouse groups. To this end, we performed immunohistochemical (IHC) staining for IL-17. As can be seen in Fig. 4a, high expression of IL-17 (brown-colored tissue staining) was observed in group II (mice with polybacterial infection and CII-induced arthritis). In contrast, in group IV (sham infection), group I (polybacterial infection only), and group III (sham infection and mice with collagen II-induced arthritis) there was much lower abundance of this cytokine in joint tissue. Immunofluorescence-based quantification (Fig. 4b) indicated that in group II 16 % of joint tissue cells were positive for IL-17 (red staining), compared to only 2.2 % in group I, and 1.3 % in group IV. As could be expected, mice with active CIA (group III) had a higher percentage of IL-17-expressing cells (8 %); this figure however, was much lower than in group II (16 %).

### Identification of oral bacteria in remote tissues

To investigate whether bacterial tissue dissemination takes place in this model, gingivae, heart, aorta, liver, pancreas, spleen, kidney, and lung were harvested after 24 weeks of infection and were examined for the presence of *P. gingivalis*, *T. denticola*, and *T. forsythia* genomic DNA by PCR using 16S rRNA gene species-specific PCR primers [17, 18, 20, 21]. As can be seen in Table 3, genomic bacterial DNA was identified in the heart, liver, kidney, and lungs of polybacterial-infected mice without CIA, and in mice infected with CIA. Among the three periodontal pathogens used for infection, there was more systemic spread of *P. gingivalis* and *T. denticola* than of *T. forsythia* (Table 3). Importantly, on FISH analysis we observed *P. gingivalis* in the perinuclear area of cells in infected ankle joint tissue (Fig. 4c (ii)). Taken together, our data demonstrate that oral infection with a PD-associated polybacterial consortium aggravates PD and CIA, and that *P. gingivalis* can be found in the inflamed joints of mice with bacterial infection-aggravated CIA, suggesting that *in situ* presence of such bacterial antigens might contribute to PD-associated arthritis.

**Table 3:** PCR detection of *P. gingivalis, T. denticola* and *T. forsythia* genomic DNA in infected B10.RIII mice tissues

Group	Polybacterial infection	Positive systemic tissue samples					
		Heart	Liver	Spleen	Kidney	Lung	Knee joint
		*n* = 10	*n* = 10	*n* = 5	*n* = 10	*n* = 10	*n* = 10
I	*Pg/Td/Tf*	4/3/1	2/3/1	0/0/0	5/4/2	2/0/0	0/0/0
II	*Pg/Td/Tf +* collagen	5/2/0	3/2/1	0/0/0	4/5/2	3/1/0	2/0/0
III	Collagen control	0/0/0	0/0/0	0/0/0	0/0/0	0/0/0	0/0/0
IV	Sham infection	0/0/0	0/0/0	0/0/0	0/0/0	0/0/0	0/0/0

After 24 weeks of gingival infection, heart, liver, spleen, kidney, lung, and hind limb ankle joint tissues were harvested from B10.RIII mice and extracted genomic DNA were subjected to 40 cycles of PCR analysis using species-specific primers for the three oral bacteria *P. gingivalis (Pg), T. denticola (Td), and T. forsythia (Tf).* The numbers indicated with forward slash correspond to the number of mice positive for *Pg/Td/Tf* genomic DNA, respectively

## Discussion

It has long been observed that patients with PD are at a higher risk of developing RA [3, 33], and patients with RA have increased likelihood of suffering from PD [34, 35]. However, the mechanistic basis of this association remains unclear. PD is triggered by bacterial gingival infection. Therefore, the realization that PD and RA share similar risk factors and pathological pathways, and the fact that effective PD treatment is associated with reduced severity of RA [36], prompted us to investigate the role of bacteria as a potential link between the two diseases. In contrast to other in vivo models of PD-associated arthritis, which are induced by infection with a single bacterial species, we studied the effect of polymicrobial dysbiotic bacterial interactions, involving a synergistic polybacterial infection. Our data show that this infection protocol required a shorter PD induction period and resulted in conclusive evidence of aggravation of CIA in B10.RIII mice. The protocol used here, involving chronic recurrent gingival infection with major periodontal bacteria, was highly efficient in adherence and colonization of infected mice with all three periodontal pathogens. This enhanced colonization was associated with a strong humoral immune response with production of IgG and IgM antibodies against the periodontal pathogens. Further, we observed significant alveolar bone resorption in polybacterial-infected mice, with or without concomitant CIA. Thus, the polybacterial colonization protocol described here produced synergistic induction of PD in B10.RIII mice.

Importantly, induction of PD by polybacterial infection facilitated CIA, as evidenced by earlier arthritis onset and a more severe arthritic process, including increased inflammatory cell infiltration and pannus formation. In addition, in vivo tomographic analysis using an MMP3 probe corroborated the induction of inflammation and enhanced severity of CIA in mice infected with polybacterial inocula compared to uninfected mice with CIA. Interestingly, the in vivo imaging data were corroborated by correspondingly increased MMP3 serum levels, consistent with our observation that polybacterial-infected mice with CIA developed more severe clinical arthritis.

Cytokines play a crucial role in the pathophysiology of RA as pro-inflammatory cytokines such as TNFα, IL-1, and IL-17 stimulate inflammation and degradation of bone and cartilage. Th17 lymphocytes and IL-17 have been recognized as essential mediators of cartilage and bone destruction. The number of Th17 cells is increased in the early stages of the disease and in active RA [37–39]. The mechanisms behind the factors promoting the Th17 differentiation in RA are poorly understood. With the significant role of pathogen-mediated TLR activation in shaping the T cell response, we reasoned that the interaction between PD and RA may in part be a direct result of skewing the Th17 cell balance. The aim of the current study was to investigate the influence of periodontitis on clinical severity and specific histopathologic features of T cell-dependent experimental arthritis. IL-17, in turn, is critical in stimulating the release of TNF-α and chemokines by joint tissues in arthritis [32].

Our IHC and immunofluorescence data supports the increased expression of IL-17 after infection with polymicrobial periodontal microflora followed by collagen administration. These observations support the hypothesis that IL-17 induces receptor activator of NF-*k*B ligand (RANKL) expression that is vital for osteoclastogenesis and bone resorption. The observations in the current study further authenticate the notion postulated by previous groups about the promotion of inflammation and the catabolic effects of IL-17 on cartilage and bone leading to the propagation of arthritis.

Finally, the identification of *P. gingivalis* in joint tissues of mice with CIA challenged with polybacterial inocula suggests that target-tissue-disseminated bacteria may contribute to the local inflammatory process, thereby augmenting the severity of arthritis in these mice. As the degree of severity, incidence, and levels of inflammatory biomarkers were all increased in polybacterial-infected mice with CIA, it is tempting to speculate that these target-tissue-seeded bacteria may be at least partly responsible for the observed association between PD and arthritis. Tissue dissemination of oral pathogens has been implicated in atherosclerotic vascular disease [40], another PD-associated condition. In that condition, it has been proposed that periodontal bacteria invade the bloodstream after various manipulations such as dental procedures or tooth brushing. According to this proposed mechanism, such bacteria are deposited in the atheromatous plaque and contribute to disease pathogenesis. It is worth noting, however, that recovery of viable bacteria from atheroma tissue cultures has been difficult, suggesting that active plaque infection by live bacteria is unlikely to be the causative mechanism. Instead, bacterial antigen-triggered stimulation of biochemical pathways or the immune system seems a more likely explanation.

Analogous to atherosclerotic vascular disease, in RA it has long been speculated that the disease may be triggered by indolent joint tissue infection. These hypotheses have been largely refuted based on the failure to consistently recover viable microorganisms from synovial tissues; however the possibility that non-viable microbial antigens might contribute to the inflammatory process in arthritis has remained a plausible hypothesis [41, 42]. For example, based on close similarities between their respective signaling pathways, we have previously proposed that the known arthritogenic effect of the bacterial antigen muramyl dipeptide (MDP) might be due to its ability to functionally mimic the shared epitope ligand [43]. We have pointed out that MDP, a building block of the bacterial cell wall and a potent immune adjuvant, displays many functional similarities to the SE ligand, including production of IL-6, TNFα, nitric oxide, reactive oxygen species, activation of osteoclasts, Th17 polarization and, importantly, facilitation of inflammatory arthritis in rodents [6, 43–46]. Obviously, the definitive molecular mechanism by which synovial tissue-seeded *P. gingivalis* contribute to the severity of arthritis needs to be examined experimentally.

## Conclusions

In summary, the findings reported herein suggest that oral bacteria play a significant role in augmenting autoimmune arthritis. Our observations support the two-hit model [47] whereby periodontopathic subgingival bacteria provide the first hit on the host immune system, leading to periodontal disease, while a second hit generated as a result of chronic inflammatory conditions in the host will eventually produce irreversible tissue damage leading to arthritis in the human host. As this model simulates the natural course of infection of oral pathogens with the naturally occurring periodontal disease in humans, it provides relevant insights into the pathogenesis of RA and could conceivably open the door to identification of new therapeutic strategies.

## Abbreviations

ABR, alveolar bone resorption; CII, collagen type II; CFA, complete Freund’s adjuvant; CIA, collagen-induced arthritis; CMC, carboxymethylcellulose; DAPI, 4′ 6-diamidino-2-phenylindole; ELISA, enzyme linked immunosorbent assay; FISH, fluorescence *in situ* hybridization; H&E, hematoxylin and eosin; IFA, incomplete Freund’s adjuvant; IgG, immunoglobulin G; IgM, immunoglobulin M; IHC, immunohistochemistry; IL17, interleukin-17; MDP, muramyl dipeptide; MMP3, matrix metalloproteinase 3; PCR, polymerase chain reaction; PD, periodontal disease; *Pg*, *P. gingivalis*; RA, rheumatoid arthritis; RANKL, receptor activator of NF-kB ligand; SEM, standard error of the mean; *Td*, *T. denticola*; *Tf*, *T. forsythia*; Th, T helper; TNF-α, tumor necrosis factor-alpha

## Additional file

Additional file 1: Figure S1. Periodontal infection induces active inflammation in polymicrobial infected + CII immunized mice. Polymicrobial-infected + CII immunized mice, *left* metatarsal tissue H&E staining showing active inflammation with infiltration of neutrophils and macrophages. **A** × 1 magnification, **B** × 2 magnification, **C** × 10 magnification (*top panel*). *I*, *II*, *III*, *IV*, and *V* are digits. CII-immunized mice metatarsal tissue H&E staining showing minimal inflammation (**A** × 1, B × 2, and **C** × 10 magnification (*bottom panel*)). *N* = 6 in each group. (TIF 2050 kb)
