# Supplementary figures and images for: Periodontal bacterial colonization in synovial tissues exacerbates collagen-induced arthritis in B10.RIII mice
Source: Arthritis Res Ther. 2016 Jul 12;18:161. doi: 10.1186/s13075-016-1056-4 (PMC4942913; doi:10.1186/s13075-016-1056-4)

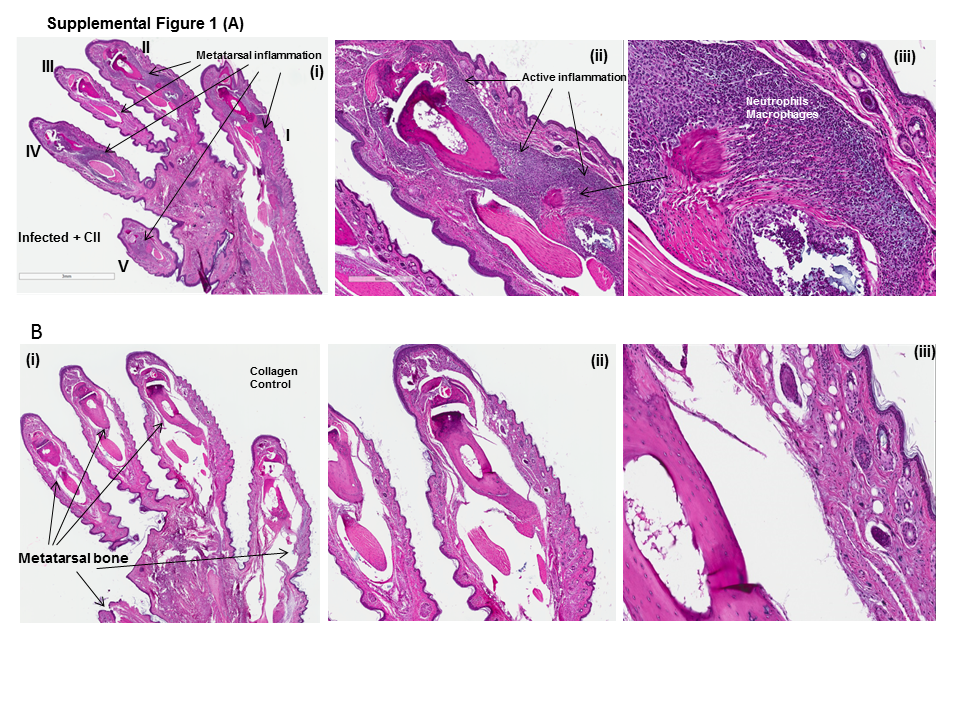

Supplement: Additional file 1: Figure S1. — Periodontal infection induces active inflammation in polymicrobial infected + CII immunized mice. Polymicrobial-infected + CII immunized mice, left metatarsal tissue H&E staining showing active inflammation with infiltration of neutrophils and macrophages. A × 1 magnification, B × 2 magnification, C × 10 magnification (top panel). I, II, III, IV, and V are digits. CII-immunized mice metatarsal tissue H&E staining showing minimal inflammation (A × 1, B × 2, and C × 10 magnification (bottom panel)). N = 6 in each group. (TIF 2050 kb) [file 13075_2016_1056_MOESM1_ESM.tif]
